# Supplementary material for: AutoPCR: automated phenotype concept recognition by prompting
Source: Bioinformatics. 2026 Jul 7;42(Suppl 1):btag304. doi: 10.1093/bioinformatics/btag304 (PMC13340160; doi:10.1093/bioinformatics/btag304)
Supplement: btag304_Supplementary_Data [file btag304_supplementary_data.pdf]

Supplementary Material

Table 1 Document-level results on three datasets. AutoPCR is the highest in average F1 and F1 ranking.

Method		BIOC-GS			GSC-2024			ID-68			Avg. F1	Avg. Rk.
		P	R	F1 (Rk.)	P	R	F1 (Rk.)	P	R	F1 (Rk.)		
Dictionary-Based	NCBO	82.64	45.70	58.85 (8)	**99.33**	52.26	68.49 (7)	88.75	64.69	74.84 (7)	67.39	7.33
	OBO	73.93	42.72	54.15 (10)	85.98	52.40	65.12 (8)	82.84	60.28	69.78 (9)	63.01	9.00
	ClinPhen	64.78	51.16	57.17 (9)	86.20	44.99	59.12 (10)	74.96	61.92	67.82 (10)	61.37	9.67
	FastHPOCR	70.92	59.77	64.87 (5)	95.15	77.61	**85.49** (1)	87.75	71.38	78.72 (3)	76.36	3.00
Neural	NCR	65.54	57.62	61.32 (7)	81.88	73.09	77.24 (6)	79.18	77.68	78.42 (4)	72.33	5.67
	PhenoTagger	71.33	65.89	68.50 (2)	87.57	76.62	81.73 (4)	84.16	72.38	77.83 (5)	76.02	3.67
	PhenoBERT	76.53	53.97	63.30 (6)	90.33	73.87	81.27 (5)	**94.11**	**78.56**	**85.64** (1)	76.74	4.00
	PhenoTagger++	69.53	64.24	66.78 (4)	89.38	76.70	82.55 (3)	79.81	72.76	76.12 (6)	75.15	4.33
Prompt-Based	Vanilla	4.42	3.48	3.89 (11)	18.25	9.75	12.71 (11)	20.04	11.98	15.00 (11)	10.53	11.00
	REAL	76.47	60.27	67.41 (3)	80.21	54.66	65.02 (9)	76.64	66.20	71.04 (8)	67.82	6.67
	AutoPCR	**84.57**	**67.44**	**75.04** (1)	89.36	**78.32**	83.48 (2)	84.33	76.71	80.34 (2)	**79.62**	**1.67**
	$AutoPCR_{FT}$	83.94	65.18	73.38	91.62	76.69	83.50	88.77	77.05	82.49	79.79	—

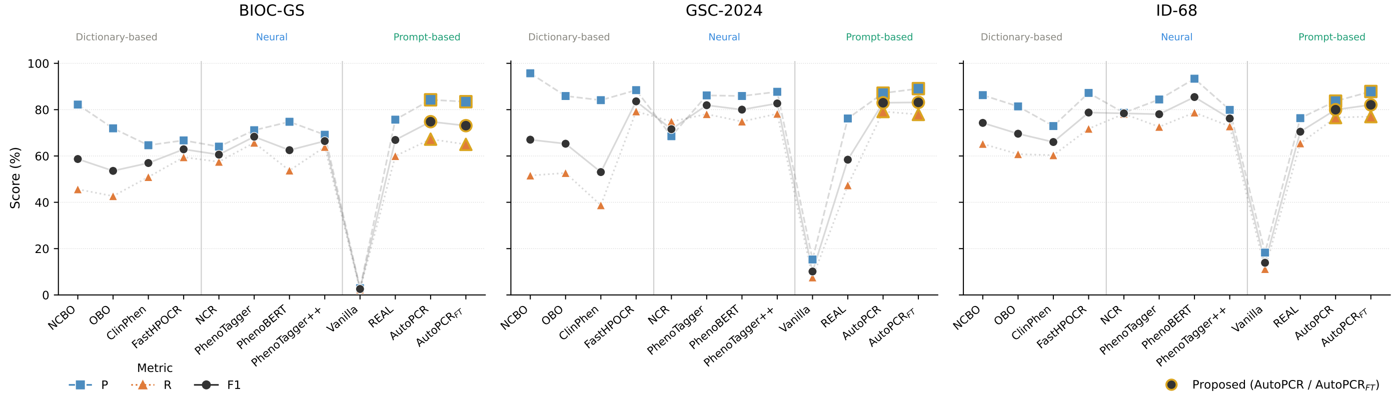

Figure 1 Visualization of mention-level results in Table 1 — precision (P), recall (R), and F1 across all methods and datasets.

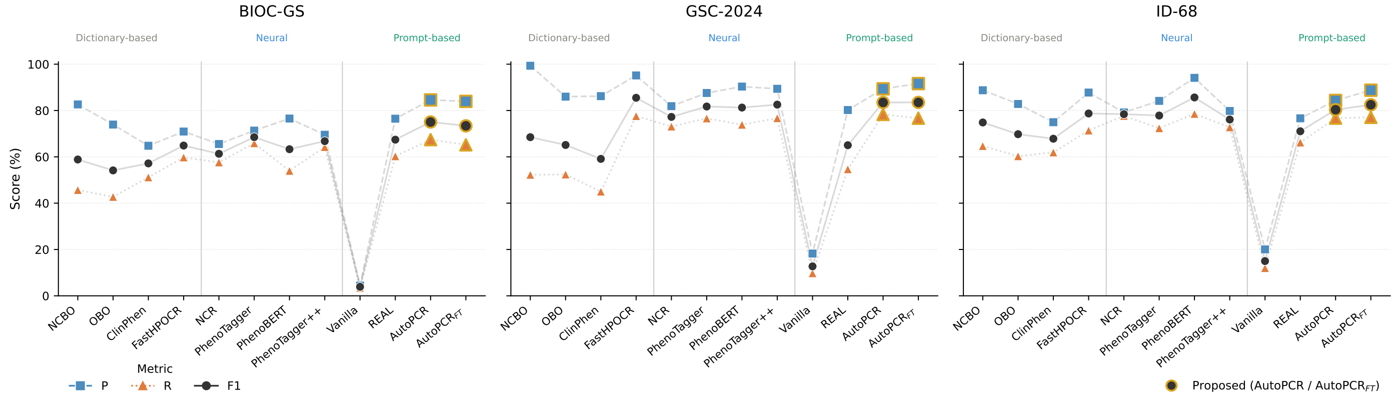

Figure 2 Visualization of document-level results in Supplementary Table 1 — precision (P), recall (R), and F1 across all methods and datasets.

Table 2 Performance of AutoPCR with various open-source and proprietary LLM backends.

	LLM Backend	BIOC-GS		GSC-2024		ID-68		NCBI		Avg. mF1	Avg. dF1
		mF1	dF1	mF1	dF1	mF1	dF1	mF1	dF1		
Open-Source	Llama-3.1-8B-Instruct	70.86±0.32	71.06±0.32	79.93±0.08	79.83±0.10	78.50±0.13	78.74±0.12	69.75±0.04	65.62±0.04	74.76±0.14	73.81±0.15
	Llama-3.3-70B-Instruct	72.63±0.19	72.85±0.20	82.33±0.04	82.72±0.05	79.20±0.11	79.56±0.13	71.00±0.02	68.07±0.14	76.29±0.09	75.80±0.13
	Llama-4-Scout-17B-16E-Instruct	72.41±0.25	72.70±0.25	82.14±0.22	82.25±0.31	**80.90±0.12**	**81.16±0.11**	72.93±0.21	70.74±0.22	77.09±0.20	76.71±0.23
	Qwen3-30B-A3B-Instruct-2507	72.60±0.03	72.81±0.03	81.71±0.01	82.03±0.00	78.73±0.00	79.07±0.00	71.36±0.02	68.84±0.00	76.10±0.01	75.69±0.01
	Qwen3-Next-80B-A3B-Instruct	**74.81±0.13**	**75.04±0.13**	82.97±0.09	83.48±0.11	79.98±0.11	80.34±0.11	**73.15±0.18**	**71.93±0.34**	**77.73±0.13**	**77.70±0.17**
	DeepSeek-V3.1	73.66±0.21	73.88±0.21	**83.15±0.03**	**83.80±0.09**	80.14±0.07	80.42±0.07	72.47±0.02	71.21±0.08	77.35±0.08	77.33±0.11
	Kimi-K2-Instruct-0905	72.77±0.12	72.98±0.12	83.00±0.25	83.70±0.31	79.33±0.14	79.74±0.17	71.46±0.37	68.70±1.23	76.64±0.22	76.28±0.46
Proprietary	GPT-4o-mini	73.95±0.11	74.18±0.11	82.71±0.09	83.04±0.10	79.36±0.10	79.63±0.11	71.54±0.05	69.05±0.07	76.89±0.09	76.47±0.10
	GPT-4.1-nano	72.55±0.24	72.76±0.24	81.92±0.06	81.93±0.10	79.27±0.12	79.55±0.13	71.06±0.10	68.54±0.15	76.20±0.13	75.69±0.16
	GPT-4.1-mini	73.66±0.07	73.88±0.07	82.67±0.03	82.90±0.02	79.18±0.09	79.55±0.12	71.79±0.09	68.80±0.09	76.83±0.07	76.28±0.07
	GPT-4.1	74.41±0.19	74.63±0.19	83.12±0.07	83.64±0.11	79.46±0.05	79.84±0.05	72.28±0.06	69.91±0.17	77.32±0.09	77.00±0.13

1 Implementation details of embedding model ablation

Variant 3a uses the untuned PubMedBERT as the retrieval backbone in place of SapBERT. Since the cosine similarity score distribution of PubMedBERT differs substantially from that of SapBERT, tuning τ_1 and τ_2 over the same grid as AutoPCR ($\tau_1 \in \{0.9, 0.925, 0.95, 0.975, 1\}$, $\tau_2 \in \{0.8, 0.825, 0.85, 0.875, 0.9\}$) yields degenerate performance. Instead, we derive τ_1 and τ_2 for PubMedBERT by matching the same similarity score routing outcome as AutoPCR, i.e., preserving the identical partition of entities into high-confidence, LLM-delegated, and filtered groups. The same procedure is also applied to Variant 3b. The corresponding thresholds and routing statistics are provided in Tables 3 and 4, respectively.

Table 3 Confidence thresholds τ_1 and τ_2 for Variant 3a (PubMedBERT) and Variant 3b (`text-embedding-ada-002`), derived by matching AutoPCR's routing partition on each dataset.

Variant	Dataset	τ_1	τ_2
3a (PubMedBERT)	BIOC-GS	0.9821309447288513	0.9681853652000427
	GSC-2024	0.9814723134040833	0.968943178653717
	ID-68	0.9853435158729553	0.970593273639679
3b (`text-embedding-ada-002`)	BIOC-GS	0.9562911987304688	0.9340839982032776
	GSC-2024	0.9612974226474762	0.9322277903556824
	ID-68	0.9669841527938843	0.9363335072994232

Table 4 Routing statistics for AutoPCR (SapBERT), used as the reference partition for Variants 3a and 3b. High-conf.: $\mathrm{sim} \geq \tau_1$; LLM: $\tau_2 \leq \mathrm{sim} < \tau_1$; Filtered: $\mathrm{sim} < \tau_2$.

Dataset	High-conf. ($\geq \tau_1$)	LLM ($[\tau_2, \tau_1)$)	Filtered ($< \tau_2$)
BIOC-GS	444	431	2,330
GSC-2024	1,045	1,080	20,756
ID-68	556	422	5,134
